# Living with chronic pain: Patients' experiences with healthcare services in Norway

**DOI:** 10.1002/nop2.160

**Published:** 2018-05-17

**Authors:** Kine Gjesdal, Elin Dysvik, Bodil Furnes

**Affiliations:** ^1^ Faculty of Health Sciences University of Stavanger Stavanger Norway

**Keywords:** chronic pain, Norway, nurses, nursing, patient experiences, person‐centred care

## Abstract

**Aim:**

To explore the experiences with healthcare received by people living with chronic nonmalignant pain in Norway.

**Design:**

A descriptive and explorative qualitative design.

**Methods:**

A total of 18 individual semistructured interviews was conducted in 2015. Qualitative content analysis was applied.

**Results:**

The findings revealed challenges related to a multifaceted pain condition. Participants described interactions with a supportive health care where being listened to, believed in and experiencing mutual trust were emphasized. When interactions with healthcare professionals made the participants feel insignificant, they found it difficult to express their needs, which seemed to reinforce practical difficulties and unfulfilled expectations and make them lose hope in their recovery. This implies the importance of a holistic understanding of and support for more person‐centred practice to accommodate patients' expectations and expressed needs. Here, the nurses have an essential role in having a positive impact on future healthcare services.

## 1 INTRODUCTION

Chronic primary pain is described as pain in one or more anatomic regions that persists or recurs for longer than 3 months. It is associated with significant emotional distress or significant functional disability that cannot be better explained by another chronic pain condition (Adler, 2006). This is a phenomenological definition, because the aetiology of many forms of chronic pain is unknown (Adler, 2006). Chronic pain is a serious problem globally and is likely to increase as the population ages (Elzahaf, Tashani, Unsworth, & Johnson, 2012). Estimates suggest that 20% of adults suffer from pain worldwide and that 90% of individuals diagnosed with chronic pain had been suffering for more than 2 years (Breivik, Collett, Ventafridda, Cohen, & Gallacher, 2006). Being undertreated contributes to a substantial burden of suffering, care and societal costs (Breivik, Eisenberg, & O`Brien, 2013; Elzahaf et al., 2012). Chronic pain should therefore receive greater attention as a global health priority. International resolutions have already declared adequate pain therapy to be a human right (IASP, 2010; WMA, 2011) and this is where registered nurses can contribute to holistic care.

## 2 BACKGROUND

Chronic pain management, as with chronic disease management in general, is conducted by the ill persons themselves, in the context of their everyday life (Barlow, Wright, Sheasby, Turner, & Hainsworth, 2002; Lawn, McMillian, & Pulvirenti, 2011). Interactions between patients and their healthcare providers are thus critically important for information exchange, decision‐making and moral support (Thorne, Harris, Mahoney, Con, & McGuinnes, 2004). Patients with severe and recurrent pain are usually referred to interdisciplinary pain clinics where they are examined and recommended a rehabilitation treatment plan (Lehti, Fjellman‐Wiklund, Stålnacke, Hammarstom, & Wiklund, 2016). Interdisciplinary pain management often involves nurses, physicians, physiotherapists and psychologists. Rehabilitation models based on a common philosophy, constant communication and the patient's active involvement are more successful than other rehabilitation models (Bosy, Etlin, Corey, & Lee, 2010; Gatchel, McGeary, McGeary, & Lippe, 2014; Merrick, Sundelin, & Stålnacke, 2012).

However, because of the limited number of physicians who are pain specialists, people with chronic pain are still treated mainly in primary health care (Gatchel et al., 2014; Harle et al., 2015). General practitioners often report frustration when caring for patients with chronic pain because of time constraints and their limited expertise in pain care (Gatchel et al., 2014). An estimated 40%–60% of patients with chronic pain experience unsatisfactory management of their pain condition (Breivik et al., 2006). People with chronic pain often have little observable physical pathology or adverse laboratory findings, so treatment can be challenging to manage in a medical context (Bendelow, 2013). A biopsychosocial approach could be a more appropriate way of understanding the complexity of chronic pain and its treatment. The biopsychosocial model is based on a holistic understanding of illness, where pain is best viewed as the result of a complex interaction of physical, cognitive, emotional, behavioural and social factors (Engel, 1977; Turk, 2003). Studies have shown that greater hope and acceptance are associated with less psychological distress, functional disability and pain (Creamer et al., 2009; Peleg, Barak, Harel, Rochberg, & Hoofien, 2009). Although there are numerous conceptualizations of hope, there is agreement on its essential characteristics: coping, being future oriented and multidimensional (Raleigh, 2000). Pain acceptance implies accepting what cannot be changed, reducing unsuccessful attempts at eliminating pain and engaging in meaningful activities despite pain (Vowles, McCracken, & O'Brien, 2003). Thus, the unique way of pain acceptance and hope contribute to individuals' adjustment to chronic pain remains to be determined (Wright et al., 2011).

Pain is a personal experience where the patient's perspective needs to be central (Jonsdottir, Gunnarsdottir, Oskarsson, & Jonsdottir, 2016). The World Health Organization (WHO) emphasizes the importance of person‐centred care (PCC) to improve health outcomes and increase well being (WHO, 2013). PCC focuses on the patient's personal needs, beliefs, preferences and experiences so that the patient become central to the care and nursing process (McCormack & McCance, 2017). This means putting the patient's expressed needs above those identified as priorities by healthcare professionals. McCormack and McCance define person‐centred practice as:

“an approach to practice established through the formation and fostering of healthful relationships between all care providers, service users and others significant to them in their lives. It is underpinned by values of respect for persons, individual right to self‐determination, mutual respect and understanding. It is enabled by cultures of empowerment that foster continuous approaches to practice development”. (McCormack & McCance, 2017, p. 3).

Recent research on chronic pain management has focused on measuring performance and effectiveness. Less attention has been paid to patients' expectations of and experiences with healthcare services. The patient experience refers to the quality and value of all interactions in the entire duration of the patient‐provider relationship (Wolf, Niederhauser, Marshburn, & LaVela, 2014). This means that the patient experience represents a continuum of care, from the first phone call to the patients being discharged. A deeper knowledge of patients' point of view has the potential to help transform health care for the better (Green & Hibbard, 2012; Richards, Coulter, & Wicks, 2015). Therefore, the aim of this study was to explore the experiences with health care received by people living with chronic nonmalignant pain in Norway. Two research questions were formulated:

- How do patients with chronic pain experience healthcare services?
- What expectations do the patients express in relation to their health care?

## 3 THE STUDY

### 3.1 Design

We used a qualitative approach with a descriptive and explorative design. A total of 18 semistructured interviews was conducted to capture the individuals' experiences. Sixteen women and two men from across Norway participated in this study.

### 3.2 Sample selection

Individuals with chronic nonmalignant pain were recruited in collaboration with a Norwegian patient organization for people living with chronic pain. Consistent with the guidelines of the patient organization, we released information about our study and our contact information. This information was published in the patient organization's official website, on its Twitter feed and a notice was placed in its printed magazine. People who were willing to participate emailed a request and the first author (KG) responded within 1 week by email or telephone. Thereafter, the participants were emailed information about the study along with inclusion criteria and an informed consent form. If they still wished to participate, KG made sure that the participants met the following inclusion criteria: age 18–67 years, nonmalignant pain >6 months, the pain condition as a primary disorder and living at home (outpatients). An appointment for conducting the interview was then made. A total of 25 people were contacted; six were excluded due to age and/or because they were suffering from a malignant pain condition and one withdrew in advance of the interview for an unknown reason. Thus, 18 patients (16 women and 2 men, aged 18–67 years) with pain conditions such as fibromyalgia, muscular pain and chronic nonmalignant pain were included (Table 1).

**Table 1:** Sample characteristics (*N* = 18)

Participants	Age	Pain area	Received health care
Male	47	Low back	GP, Pain clinic, Rehabilitation stay
Male	42	Low back	GP, Pain clinic
Female	64	Back	GP
Female	38	Neck/shoulders	GP, Pain clinic, Rehabilitation stay
Female	61	Muscular	GP, Pain clinic, Rehabilitation stay
Female	42	Neck/shoulders	GP, Rehabilitation stay
Female	65	Muscular	GP
Female	50	Muscular	GP, Pain clinic, Rehabilitation stay
Female	45	Migraine	GP, Rehabilitation stay
Female	22	Muscular	GP
Female	37	Low back	GP, Pain clinic
Female	42	Neck/shoulder	GP, Pain clinic
Female	28	Pelvis	GP, Pain clinic, Rehabilitation stay
Female	50	Migraine	GP
Female	52	Neck/shoulders	GP, Pain clinic, Rehabilitation stay
Female	28	Muscular	GP, Pain clinic
Female	45	Low back	GP
Female	18	Knee/calf	GP
	Mean age 43		

GP, general practitioner.

### 3.3 Data collection and analysis

The interviews were conducted using a semistructured interview guide and lasted 55–75 min. The researcher provided some structure based on the interview guide but allowed rooms for the participants to offer more spontaneous descriptions and narratives. The topics in the interview guide were: everyday life with chronic pain, care‐specific challenges and experiences with offered health care. Each participant was given a choice to conduct the interview at his or her home or elsewhere (e.g. a conference room). Ten participants were interviewed at their home, six patients at a conference room in a hotel and two interviews were conducted in a conference room at the first author's workplace. All participants agreed to have the interview tape‐recorded. The interviews were analysed using qualitative content analysis as presented by Graneheim and Lundman (Graneheim, Lindgren, & Lundman, 2017; Graneheim & Lundman, 2004). The analytical process transpired in six stages as outlined in Table 2. Qualitative content analysis focuses on subject and context and emphasizes variation, such as similarities and differences between parts of the text (Graneheim et al., 2017). Consistent with a hermeneutic phenomenological point of view, we strived to be close and connected to the study participants to elicit meanings in the data using various degrees of interpretation. An overview of subthemes abstracted into themes is shown in Table 3.

**Table 2:** Stages of the analytic process

1 Open reading	Read each script several times to gain an impression of what was being said.
2 Identifying meaning units	Divided into meaning units where each meaning unit is related to the same central content.
3 Condensed meaning unit	Condensed the meaning units into a more formalized and written style.
4 Creating codes	Labelled the condensed meaning units with codes
5 Sorted codes and abstracted into subthemes	Sorted the codes and abstracted them into 8 subthemes. Continuously discussed tentative subthemes with research fellows.
6 Formulating into a latent theme	Formulated the latent content of the sub‐themes into themes in collaboration with research fellows.

**Table 3:** Overview of the subthemes abstracted into two themes

Subthemes	Themes
Being taken seriouslyGetting practical supportGetting emotional supportHaving belief in the future	Feeling acknowledged as a person in the healthcare services
Not being taken seriouslyHaving practical difficultiesHaving unfulfilled needs and expectationsLosing hope	Feeling neglected as a person in the healthcare services

### 3.4 Rigour

To ensure a trustworthy study, we used the credibility, transferability, dependability and conformability criteria as presented by Lincoln and Guba (1985). To enhance the credibility of the data collection, the same researcher conducted all interviews. The findings were critically interpreted to create a comprehensive understanding and were elaborated in the light of existing literature and the applied biopsychosocial model. The inclusion of 18 individuals from across Norway with various pain conditions and various health care experiences reinforced the credibility, as the participants offered comprehensive descriptions of the issues examined. Table 2 provides a detailed description of the analytical steps. In addition, Table 3 shows an overview of the subthemes abstracted into themes. Overall, this may strengthen the credibility and dependability of our findings. The transferability of our findings is enhanced by using quotations from the data along with detailed descriptions of the participants. Our findings may be transferable to other professionals or people in similar situations by considering the patients' culture and context, as well as methods of data collection and analysis. Recognition of the shortcomings in the study to ensure conformability is outlined in the section*:* Strengths and Limitations.

### 3.5 Ethics

The study was approved by the Regional Committees for Medical and Health Research Ethics, Norway (Project number 2014/2165). Every participant provided informed written consent before the interview. All participants were informed both in writing and verbally about their rights to withdraw at any time and that their participation was anonymous.

## 4 RESULTS

The participants had experiences with primary health care, from specialist care settings including pain clinics and from stays at rehabilitation units. This made it difficult to distinguish among the different types of healthcare providers or professions involved. The findings are therefore presented with a focus on healthcare professionals in general. Through the analysis, two themes were developed: “Feeling acknowledged as a person in the healthcare services” and “Feeling neglected as a person in the healthcare services”. In the following, the two themes are presented, and quotes are provided to give the participants a clear voice.

### 4.1 Feeling acknowledged as a person in the healthcare services

Feeling acknowledged as a person was characterized by being taken seriously, getting practical and emotional support and having belief in the future. Being taken seriously in interactions with healthcare professionals was described in different ways. Among these, being listened to, believed in and experiencing mutual trust were emphasized:

He [the GP] took the time and talked to me, not just read my journal. He wanted to hear it [anamnesis] from me.

The participants experienced that their health‐related problems were addressed appropriately after a supportive meeting with healthcare professionals. Feeling significant and being listened to encouraged the sense of being in a partnered relationship. The participants also noted several practical needs related to their pain condition and experienced important help especially from their general practitioner (GP) to coordinate information from different healthcare providers. The healthcare professionals usually hold the key to accessing practical support in terms of coordinating medical information and referring to specialist care or staying at rehabilitation units:

Finally, I've got a doctor who is wonderful, it's great. We work as a team.

My GP knew about this rehabilitation unit and thought of me, I'm so grateful for that.

The participants' reiterated the importance of healthcare providers in collaborating with other specialists to give updated care and at the same time search for available therapies. When a patient's GP cooperated with the local pain clinic, this was experienced as especially fruitful. The participants also described a pain management programme offered at the pain clinic or at the rehabilitation unit as valuable, in teaching them ways of managing their chronic pain in everyday life:

I learnt some mental tools after a visit to the pain clinic, but it's still me who has to do the job. I'm proud of what I've done.

The need to attend a rehabilitation programme was emphasized. Pain rehabilitation taught people to be patient, to be careful with physical activity and to know their body much better. The participants experienced that their condition was properly addressed at the rehabilitation unit. These findings indicate that living with chronic pain can be very demanding and that emotional support from healthcare providers is essential. The participants communicated a sense of a strong relationship and good teamwork with their healthcare professionals. Continuity with professionals who knew their life story was also important. The participants experienced emotional support in terms of supportive conversations:

My GP sees the whole picture, no matter what the problem is, she has always been there for me.

I have good conversations with my GP and also with my physician, which helps me to deal with it and accept it, that this is life at the moment.

Being taken seriously by healthcare professionals and receiving practical and emotional support seemed to affect their future hopes, so hope and acceptance were essential. Even though they had accepted their pain condition, they maintained some hope of recovery:

I'm not giving up. There's a difference between hoping and accepting, I've accepted that this is life at the moment, these are the cards I've been given.

I've accepted my situation as it is today, but no one can take away my hope, that 1 day there may be something that can help me, although this does not exist today.

Participants talked about some supportive meetings or that they perceived at least some parts of the received health care as being supportive. However, all participants experienced inadequate meetings with an often impersonal healthcare system. For some, this was the reality in their interactions in primary care, in specialized care settings and at rehabilitation units. Thus, the following theme of “feeling neglected as a person in healthcare services” comprises our more significant findings.

### 4.2 Feeling neglected as a person in the healthcare services

Feeling neglected as a person was characterized by not being taken seriously, having practical difficulties, unfulfilled needs and expectations and by losing hope. Unsupportive encounters were characterized by a lack of recognition. The participants experienced not being believed, listened to, or respected. This gave them the impression of being written off and mistrusted by the healthcare professionals:

This terrible feeling of not being believed in and not being listened to. The only thing I have asked for is help so that I can take care of myself.

According to the participants, some healthcare professionals dismissed their illness experiences as irrelevant or nonexistent. Participants talked about meetings with different healthcare workers where they felt brushed aside and felt accused of imagining their illness, because there were no objective signs of disease. Participants who were not satisfied with their GP were afraid to look for treatment elsewhere, because they feared that their new GP would likewise neglect their diagnoses:

They don't find anything, so there is nothing wrong. I knew there was something wrong, so that message was tough for me to deal with. However, when the doctor says so, you are hitting a wall.

I've considered changing my GP, after I was disappointed at the last consultation. Then I started thinking, to begin all over again, will they believe me?

Difficult interactions with healthcare professionals were also linked to practical needs. In most cases, the participants experienced being placed on waiting lists and not receiving complete information and noted that the health care they received was impersonal. Other practical obstacles experienced were a fragmented healthcare system, where different services failed to work in tandem. Participants also complained that short consultations with their GP were poorly suited to complex diagnoses such as chronic pain:

I'm caught running between them [the GP and specialist care]; he said that and she said something else, which doesn't help much.

It's too much [the complex issues] for a doctor with only 15‐minute consultations. I bring notes about the most important issues, but we only get through the first two and then we have to postpone the rest for my next consultation. But the next time, I have new issues.

Unfulfilled needs and expectations were emphasized in many ways, with some related to a treatment that only consisted of different kinds of medication. The participants felt left alone, having to take personal responsibility for following up with prescribed medications. They pointed out the need for more information about their treatment options and rights. The participants also described experiencing insufficient benefits from rehabilitation stays, not being able to manage the time schedule and feeling worse afterward, given that the treatment was not tailored to each patient:

They have resorted to different medications; soon I'll have a pharmacy at home. They refuse to offer physiotherapy or any rehabilitation.

When I got home [from rehabilitation] I felt sicker, exhausted. I had to take breaks on my way to the toilet. After this experience, I'm afraid of doing any physical activity.

The participants described living with chronic pain as demanding and expressed feelings of hopelessness related to an inadequate healthcare system. Participants stated that their lived experience of illness was neither respected nor of any significant value in the care situation. They described this lack of recognition as destructive. Hopelessness was also related to the feeling of being a low priority in the healthcare system:

You will not be given priority since it's not fatal. But because of the mental part of it, then it could actually be fatal. You're in so much pain, that you just don't want to live anymore. But it seems like that's not important to them.

What I see, to the extent that I can be objective, is a health care that has managed to break me down.

Participants described disheartening conversations where healthcare professionals highlighted the importance of “acceptance” in such terms that they interpreted this as meaning there was no hope of making progress. The one‐sided focus of healthcare providers on the importance of acceptance—in the sense that things would never get better—was so depressing that the participants feared losing their spark of life:

It's such a shame that health care providers present as an established fact that a person with chronic pain who believes he can regain a normal life has to be “realistic”. They say you have to learn to live with it. I think this is a big mistake. The importance of accepting the situation as it is doesn't mean that you can't make any progress or that you can't reduce your pain, or be free of pain in the future. This information is absent.

They [health care workers] rationalize away hope and what gives a person the enthusiasm to try. You need something that gives you the excitement to believe that it's possible.

Several participants expressed no hope of recovery and had given up on fighting for themselves. They mentioned the importance of maintaining and respecting their future hopes of recovery in interactions with healthcare professionals.

## 5 DISCUSSION

Our aim was to explore the experiences with health care received by people living with chronic nonmalignant pain in Norway. Two themes were developed through the content analysis. The following discussion is arranged according to the dichotomous themes: “Feeling acknowledged as a person in the healthcare services” and “Feeling neglected as a person in the healthcare services” to contrast the different experiences.

Participants described some satisfactory interactions with a supportive standardized health care where they were taken seriously. Emphasis was placed on the importance of being listened to, believed in and experiencing mutual trust, when their illness was considered as valid and important. According to Cissna and Sieburg (1981), interpersonal confirmation highlights the elements of existence, confirming: “to me you exist,” “we are relating,” “to me you are significant” and “your way of experiencing your world is valid” (Cissna & Sieburg, 1981 p. 259). Although confirmation has long been identified as crucial in forming and maintaining any human relationship, it has received the most attention in clinical or psychotherapeutic settings (Cissna & Sieburg, 1981). This behaviour in a healthcare context, like our findings, could influence the interactions between patients and healthcare professionals in such a way that the patients feel valuable and thereby confirmed. Models of nursing, regardless of their philosophical underpinnings, have prioritized the importance of relationships (Watson, 1997). A qualitative systematic review investigated the patient–professional relationship and stressed the patients' need to be understood and their expectations towards health professionals for understanding their pain and life situations (Fu, McNichol, & Marczewski, 2015). The quality of these relationships is mentioned in patient satisfaction surveys as being of particular importance (Wiechula et al., 2016). As we see it, the patients' relationships with healthcare workers appears central to the quality of the patients' healthcare experience.

When the participants were taken seriously in their interactions with healthcare providers, they described increased practical support such as being referred to pain centres or stay at rehabilitation units. When their illness was validated in these interactions, they also described emotional support, which seemed to make them feel more optimistic. Patients' satisfaction with chronic pain management can be seen as the result of the match between expectations and subsequent experiences (Geurts et al., 2016). Meeting patients' expectations should result in consistency between patients' needs and healthcare delivery, resulting in greater satisfaction with care (Barbosa, Balp, Kulich, Germain, & Rofail, 2012). Satisfaction with care might increase compliance, which may then improve pain management outcome (Nicholas et al., 2011). We suggest an increased awareness in identifying the patients' needs and expectations to enhance healthful relationship during consultation.

However, it is important to stress that all participants experienced inadequate interactions with an often impersonal healthcare system. The participants described disconfirmation, based on their impression that healthcare workers invalidated their lifeworld and illness experience. The participants seemed to equate this lack of confirmation with a lack of interest. Patients want confirmation that their chronic pain is “real” and want to feel empowered through access to reliable information on best practices (Dewar, Gregg, White, & Lander, 2009). As such, an increased focus on confirmation in the dialogue between patients and healthcare workers seems vital to diagnostic and therapeutic processes as it forms the basis for this relationship.

The participants experienced lack of recognition, as they struggled to be believed and taken seriously. Unsupportive encounters in healthcare settings where the patients felt accused of exaggerating or imagining their illness due to the lack of observable signs of disease were highlighted, consistent with previous studies (Håkanson, Sahlberg‐Blom, & Ternestedt, 2010; Lehti et al., 2016). Werner and Malterud (2003) found that women with chronic pain struggle to fit in with normative biomedical expectations by trying to act credible in their encounters with healthcare providers. We agree with the argument that many of the attitudinal problems are linked to the predominance of acute care models, which dominate most healthcare levels where people with chronic illness are found (Thorne, 2006).

Chronic pain is a complex phenomenon resulting from biological, psychological and social factors, all of which are relevant to pain management (Engel, 1977). This biopsychosocial approach, presented by Engel in 1977, does not abandon the biomedical model but rather extends it (White, 2005). Although the biopsychosocial approach is widely accepted, the corresponding introduction of multidisciplinary pain clinics employing specialist treatments tailored to individual patient needs has not always followed (Kress et al., 2015). A review of stakeholder groups revealed several reasons for this, including rushed consultations and pain management having a low priority and being underresourced (Kress et al., 2015). Pain management, which should be multidimensional, often depend primarily on pharmacology, which in itself are presenting patients and the healthcare system with enormous challenges (Penney, Ritenbaugh, DeBar, Elder, & Deyo, 2016). Prescription for opioid medications for chronic pain has increased dramatically and is associated with increased opioid overdose, abuse and other harm in addition to uncertainty about long‐term effectiveness (Chou et al., 2015). Along with the rise in opioid prescribing for pain management, concerns about the limitations and efficacy of this treatment have increased. Furthermore, the clinical milieu has emphasized the importance of treatment focusing on improving patients' functioning rather than pain reduction (Ballantyne & Sullivan, 2015). We underline the importance of an increased focus on the challenging shift from cure to care in chronic pain management, as this might contribute to a more person‐centred approach to treatment earlier in each patient's trajectory.

The participants described feelings of hopelessness and resignation when experiencing unfulfilled needs and expectations. Inadequately managed pain can have adverse physical and psychosocial patient outcomes for individual patients and their families (McCormack & McCance, 2017). Not being seen and the inability to escape from pain may create a sense of helplessness and hopelessness. The participants highlighted the importance of healthcare professionals in maintaining and respecting their future hope of recovery. Hope can enable patients to cope with a stressful situation by expecting a positive outcome. In turn, because a positive outcome is expected, patients are motivated to act despite uncertainty (Rice, 2012). Recently, there has been growing recognition that positive psychological factors, for example pain acceptance, hope and optimism may be related to how individuals adjust to chronic pain (Wright et al., 2011). Studies identify the role of pain acceptance as an important predictor of pain adjustment, pain‐related disability and to less psychological distress (Elander, Robinson, Mitchell, & Morris, 2009; Goodin & Bulls, 2013). Thus, current pain practice encourages acceptance of chronic pain, rather than the ongoing search for a cure (Dewar et al., 2009).

However, present findings indicate that patients might not respond positively when told to accept their situation and live with the pain. Given this message, the participants interpret the healthcare professionals' focus on ‘acceptance’ as meaning that their search and hope for relief should end; as a result, the participants then seemed to lose hope and give up. This is in line with previous research, which revealed that women living with arthritis and fibromyalgia rejected the term ‘acceptance’ (Lachapelle, Lavoie, & Boudreau, 2008). The rejection reflects the woman's beliefs that acceptance is resignation (Lachapelle et al., 2008). According to McCracken, acceptance is not a decision or belief about pain but a process by which the individual began to make lifestyle choices that maximize their quality of life (McCracken & Vowles, 2006). As such, it is essential to develop a clear picture of the patients' values concerning their lives and how they make sense of what is happening. As we see it, a move towards person‐centred practice appears to be a promising avenue for chronic pain management, because it is essential to understand what is important to each patient.

## 6 STRENGTHS AND LIMITATIONS

The term “information power” guides sufficient sample size in qualitative studies (Malterud, Siersma, & Guassora, 2016). The size of a sample with sufficient information power depends on the purpose of the study, specific aim, use of established theories, dialogue quality and analysis strategy. Information power in this study was influenced by a specific aim (experiences with health care) with dense specificity (patients' experiences), along with the applied biopsychosocial model. The first author conducted all interviews and a thorough qualitative content analysis was performed (Graneheim & Lundman, 2004). Thus, 18 informants from across Norway included rich and nuanced descriptions of the phenomena and the data had satisfactory information power to develop valuable knowledge related to our aim.

This study has the following limitations. First, all participants were recruited from a patient organization and thereby represent those who were willing and able to join such associations. Second, the study sample had 16 women but only two men. It is uncertain whether men are less willing to talk about pain or whether men are less likely to seek health care for pain. However, this mirrors the actual distribution among those with chronic pain, where women are more often diagnosed with a chronic pain condition than men (Bartley & Fillingim, 2013). Third, we did not reveal any obvious differences in experience by age group. However, this could be an interesting dimension in further research and discussion.

## 7 CONCLUSION

Our findings stress that it is vital for individuals with chronic pain to have their illness experiences and lifeworld considered as valuable. This study provides new and extended knowledge, indicative of a need for greater recognition of the patient's lifeworld and expectations in chronic pain management settings. Our participants experienced challenges related to their multifaceted pain condition. This implies the importance of holistic understanding and support for more person‐centred practice to accommodate patients' expectations and expressed needs. Here, the nurses have an essential role in having a positive impact on future healthcare services.

## 8 CLINICAL IMPLICATIONS

Nurses should be at the forefront of achieving a biopsychosocial approach to pain management, in accordance with person‐centred care. To achieve genuine person‐centred practice, we suggest that nurses should have a leading role and pay more attention to the patients' values, expectations and expressed needs. More knowledge on person‐centred care in chronic pain management settings is required. Further research should be undertaken on individualized pain management from the points of view of both patients and professionals.

## AUTHOR CONTRIBUTIONS

KG, ED and BF designed the study. KG conducted and transcribed all interviews. KG, ED and BF analysed the data and KG drafted the manuscript. All authors contributed to editing of the final manuscript, revised it critically for scientific content, read and approved the final version.
